# Spraying humic acid regulator on cultivated *Codonopsis pilosula* (Franch.) Nannf. to improve yield of active constituents

**DOI:** 10.3389/fpls.2024.1381182

**Published:** 2024-05-30

**Authors:** Gaojian Huang, Huifeng Miao, Yaqian Chen, Ke Wang, Qiang Zhang, Zhiping Yang

**Affiliations:** ^1^ College of Resource & Environment, Shanxi Agricultural University, Taiyuan, Shanxi, China; ^2^ Shanxi Province Key Laboratory of Soil Environment and Nutrient Resources, Shanxi Agricultural University, Taiyuan, Shanxi, China; ^3^ Nitrate Fertilizer Technology Innovation Center of Shanxi Province, Shanxi Knlan Chemical Co., Ltd., Taiyuan, Shanxi, China

**Keywords:** medicinal plant, plant growth regulator, secondary metabolites, chlorocholine chloride, field experiment

## Abstract

Plant growth regulators have been used in the cultivation of medicinal plants to increase yield, but the existing regulators decreased the content of active constituents which benefit human health. Therefore, it is necessary to find a new growth regulator to achieve the win-win goal of increasing yield and improving active constituents’ accumulation. The potential of replacing chlorocholine chloride with a new humic acid-based growth regulator was evaluated by measuring the yield and active constituents’ accumulation of *Codonopsis pilosula*. Three treatments including water (CK), chlorocholine chloride (T1) and humic acid regulator (T2) were applied by foliar spraying. Among them, both chlorocholine chloride and humic acid regulator belong to biostimulant. The result showed that the root yield in T1 and T2 were significantly increased by 59.1% and 54.9% compared with CK, respectively, and there was no significant difference between T1 and T2. Compared with CK, the yields of lobetyolin, syringin and atractylenolide III of *Codonopsis pilosula* were significantly decreased by 6.3%, 7.3% and 13.0% in T1, but were significantly increased by 22.8%, 14.8% and 32.0% in T2, respectively. Redundancy analyses showed that photosynthetic rate, sucrose phosphoric acid synthetase and phosphomannomutase had higher degree of explanation for yield and quality. Linear regression results indicated that photosynthetic rate and phosphomannomutase were the main factors to affect yield and active constituents yields, respectively. In addition, the output-input ratios based on the yields of polysaccharides, lobetyolin, syringin and atractylenolide III of *Codonopsis pilosula* in T2 was significantly increased by 6.5%, 15.2%, 8.7% and 31.2% respectively as compared with T1. Overall, compared with water treatment, both chlorocholine chloride and humic acid regulator treatments can increase the root yield of *Codonopsis pilosula*. Compared with chlorocholine chloride, humic acid regulator can improve the yield of active constituents and economic benefits of *Codonopsis pilosula*. This study indicated that reasonable selection of plant growth regulators is of great significance for achieving a win-win goal of increasing the root yield and active constituents of medicinal plants.

## 1 Introduction

The active constituents in medicinal plants have therapeutic effects on diseases, which can be used as raw materials to improve human health. *Codonopsis pilosula* (Franch.) Nannf. is a medicinal plant with a long application history, which is mainly cultivated in East Asia, Southeast Asia and Central Asia (Gao et al., 2018; Bai et al., 2021). China is one of the main sowing countries with a planting area of more than 5.33×10^4^ hm^2^ (Yang et al., 2019). Studies have shown that The main active constituents of *C. pilosula* are compounds like polysaccharides, lobetyolin, syringin and atractylenolide III, which have certain pharmacological effects and are the main contributors to the efficacy of *C. pilosula*.

In recent years, domestic and foreign scholars have conducted further research on the active constituents and pharmacological effects of *C. pilosula*, and found that different active constituents can exert curative effects in vivo through different mechanisms of action. For example, polysaccharides are macromolecular compounds formed by the polymerization of multiple monosaccharide molecules through glycosidic bonds. The monosaccharides that constitute polysaccharides mainly include arabinose, glucose, galactose and mannitol (Zou et al., 2020). Zhang et al. (2017) injected pectin polysaccharides of different concentrations (0; 50; 100; 200 g mL^-1^) into spleen cells of mice and found that polysaccharides can regulate the proliferation reaction of spleen cells by regulating the percentage of T lymphocyte types and enhancing the production of proinflammatory cytokines. The effect is proportional to the concentration, indicating that polysaccharides have a regulatory effect on immunity. Lobetyolin belongs to phenylpropanoid glycosides compounds, and its molecular formula is C_20_H_28_O_8_. Xie et al. (2024) fed mice with lobetyolin of different concentrations (0.2; 0.4; 0.6 g kg^-1^) for 7 days and found that lobetyolin of 0.6 g kg^-1^ significantly reduced the abundance of intestinal flora, repaired intestinal mucosa and inhibit the entry of lipopolysaccharide into body’s circulation, and improve intestinal mucosal dysfunction. Syringin also belongs to phenylpropanoid glycosides compounds, and its molecular formula is C_17_H_24_O_9_. Lee et al. (2019) injected 0.2 mL syringin of 5 mg kg^-1^ into mice with tumorigenic breast cells and found that syringin could inhibit the growth of breast cancer cells by up regulating the expression of cystatin and DNA repair enzyme, and down regulating the expression of cyclin dependent kinase 4 and X-linked inhibitor of apoptosis, thus playing an anti-cancer role. And atractylenolide III belongs to triterpenoid compounds, and its molecular formula is C_15_H_20_O_3_. Novianti et al. (2021) treated microglia of mice with atractylenolide III (100 μM) *in vitro* and found that atractylenolide III inhibit the mRNA expression of tumor necrosis factor, interleukin and pleiotropic pro-inflammatory cytokines, and inflammatory enzyme activity in cells, indicating that atractylenolide III has anti-inflammatory effects in the peripheral nervous system. Presently the production of wild *C. pilosula* can no longer meet the demand of market due to the increasing quantity demanded by the pharmaceutical industry. This has led to a rapidly expanded acreage of cultivated *C. pilosula*. In this context, in order to obtain more yield and contents of active constituents such as polysaccharides, lobetyolin, syringin and atractylenolide III in cultivation, the application of plant growth regulators has been an important way to improve the yield and quality of medicinal plants, and thus maximizing economic benefits.

Plant growth regulators (PGRs) can increase the yield of crop and improve its quality because they have synthetic compounds with phytohormonal activity (Rademacher, 2015; Pu et al., 2018). PGRs have been widely used in agriculture, forestry, horticulture and other fields (Giannakoula et al., 2012; Huang et al., 2021; Katel et al., 2022). Researches have found that PGRs can increase yield by affecting a series of physiological and biochemical parameters such as chlorophyll content, photosynthesis and enzyme activity during the growth and development of medicinal plants (Afreen et al., 2023). In addition, PGRs can also increase contents of active constituents by regulating the activity of metabolic enzymes and gene expression during the secondary metabolism in medicinal plants (Jamwal et al., 2018). Previous research found that PGRs had different effects on various medicinal plants. For instance, the application of jasmonic acid on *Perilla frutescens* increased the content of anthocyanin by about 4.3 times compared with the control (Szymanowska et al., 2015). The application of gibberellin on peppermint significantly increased the content of menthol and menthone (Ahmad et al., 2019). But the application of chlorocholine chloride on *Salvia miltiorrhiza* significantly decreased the content of tanshinone (Lyu et al., 2023). And spraying mepiquat chloride on *Scutellaria baicalensis* leaves led to a decrease in contents of baicalin and baicalein (Hu et al., 2012). Therefore, the effects of PGRs on medicinal plants may depend on their type. For example, applying exogenous gibberellin to *Salvia miltiorrhiza* increased the content of tanshinone in roots (Yuan et al., 2008), while the application of chlorocholine chloride led to a significant decrease in tanshinone content (Lyu et al., 2023). Thus, it can be seen that in the cultivation of medicinal plants, it is necessary to select the type of growth regulators scientifically and normatively, which has a positive significance for the quality of medicinal plants.

According to their physiological functions, PGRs can be classified into growth promoter, growth retardant and growth inhibitor (Zhang et al., 2024). Among the different plant growth regulators, the “root-strengthening” growth regulator is widely used because it can significantly increase the yield of many rhizome herbs including *C. pilosula*. However, the inappropriate use of the “root-strengthening” growth regulator can deteriorate the quality of herbs. For example, spraying paclobutrazol at different concentrations (1; 5; 10; 25; 50 g L^-1^) on *Ophiopogon japonicus* resulted in a decrease in the content of saponin, and spraying with 50 g L^-1^ paclobutrazol led to the lowest contents of saponin. And the flavonoid content increased only when 1 g L^-1^ paclobutrazol was sprayed (Zhang et al., 2019). Chlorocholine chloride is the main ingredient of the “root-strengthening” growth regulator. Although the application of this agent can improve the yield, it can significantly diminish the contents of active constituents in *C. pilosula* (Liao et al., 2017). Therefore, it is essential to achieve the win-win goal of maintaining yield and increasing content of active constituents in the cultivated *C. pilosula* by developing a new plant growth regulator as a substitute for chlorocholine chloride.

Humic acid is a type of macromolecular organic compound containing aromatic structures formed by chemical reactions and microbial decomposition of animal and plant remains (Shah et al., 2018). Its basic structure is composed of aromatic and aliphatic rings connected by functional groups such as hydroxyl, carboxyl, methoxy, and carbonyl groups (De Melo et al., 2016). Humic acid fertilizer is increasingly used in agricultural production with the development of agriculture and the improvement of humic acid fertilizer production technology. Humic acid has been approved to significantly improve yield and quality of cereals, vegetables and fruits (Ibrahim and Ramadan, 2015; Hafez et al., 2021; Chen et al., 2022; Miri Nargesi et al., 2022). Studies have shown that humic acid increases yields by stimulating root growth and enzyme activity, as well as improving chlorophyll content and photosynthetic efficiency in plants (Fan et al., 2014; Chen et al., 2022). In recent years, humic acid fertilizer has been widely applied in the growing of medicinal plants. The application of humic acid water-soluble fertilizer leads to an improved yield of *Astragalus membranaceus* by 7.1% and increased content of Calycosin-7-glucoside (Guo et al., 2019). Foliage spray of humic acid on *Echinacea purpurea* can significantly increase its chlorogenic acid content (Khorasaninejad et al., 2018). However, the effects of humic acid on the yield and contents of active constituents in *C. pilosula* remained unclear, and the investigation of the differences between the effects of humic acid and chlorocholine chloride on the herb is very important for determining the possibility of replacing chlorocholine chloride with humic acid.

Therefore, we hypothesized that spraying of humic acid regulator can achieve the win-win goal of increasing the yield and contents of active constituents of *C. pilosula*. The objectives of our study were to: (1) compare the effects of chlorocholine chloride and humic acid regulator on the yield of *C. pilosula*, (2) compare the effects of chlorocholine chloride and humic acid regulator on polysaccharides, lobetyolin, syringin and atractylenolide III, and (3) evaluate the economic benefits of chlorocholine chloride and humic acid regulator.

## 2 Materials and methods

### 2.1 Description of experimental site

The experiment was conducted in Shijiapo Village, Lingchuan Country, Jincheng City, Shanxi Province, China (35°79′52″N, 113°44′74″E, 1200 m a.s.l.). The study area has a temperate continental monsoon climate, the annual precipitation is about 500 mm, while the mean annual temperature is about 7.9°C. The experimental area receives an annual sunshine duration of 2,630 h, and the average frost-free period spans around 120 days (Huang et al., 2022). The natural conditions of the experimental area are conducive to the growth of *C. pilosula*. The soil at the experimental site is cinnamon soil with uniform fertility. Before the commencement of the experiment, the baseline physicochemical properties of the soil (0 - 20 cm) at the research site were tested as follows: 35.4 g kg^-1^ organic matter, 2.11 g kg^-1^ total nitrogen, 15.90 mg kg^-1^ available phosphorus and 275.02 mg kg^-1^ available potassium with pH of 8.13.

### 2.2 Experimental materials

The robust and undamaged seedlings with consistent size and at least one terminal bud were selected for experiment. And we selected humic acid regulator and chlorocholine chloride, they belong to biostimulant. The humic acid regulator was developed by our group, which contained at least 50 g L^-1^ of humic acid as the main ingredient. The “root-strengthening” growth regulator contained 100 g L^-1^ of chlorocholine chloride commonly used by local farmers was used.

### 2.3 Experimental design

The experiment was a randomized block design with three treatments: water (CK), chlorocholine chloride (T1) and humic acid regulator (T2). Each treatment had three replicates, and the area of plots was 60 m² (10 m×6 m). Transplantation was taken place on April 5th, 2022 with a plant spacing of 25×10 cm. The planting density was 4×10^5^ plants hm^-2^, with reference to the most suitable density in the area. Before transplantion, 750 kg hm^-2^ of fertilizer was applied evenly as basal fertilize.

The regulators was applied for the first time at the initial flowering stage of *C. pilosula.* The humic acid regulator was prepared as a 0.05% solution and applied via foliar spraying for 4 times on August 4th, August 19th, September 5th, and September 20th. Chlorocholine chloride was applied twice on August 4th and August 19th as a 0.1% solution following local farmers’ routine practice. Other field management measures remained consistent among all groups. Harvesting and yield determination were conducted on November 8th after the shoots had withered. Three 2 m × 2 m quadrat of was selected randomly from each plot for harvesting. Samples were brought to the laboratory where the roots were washed with water to remove soil. Subsequently, the samples were transferred into kraft bags and dried in an oven at 105°C for 30 minutes and then at 75°C to constant weight. Finally, the samples were pulverized and kept at stored at room temperature for further analysis.

### 2.4 Determination of yield parameters and contents of active constituents

#### 2.4.1 Determination of root growth and yield

Root activity (mg g^-1^ h^-1^) was determined by the triphenyltetrazolium chloride method (Liu et al., 2008). Root dry matter weight (g plant^-1^) referred to weight of a single root after being dried to constant weight. Yield (kg hm^-2^) randomly select three 2 m × 2 m quadrat from each plot and harvest the roots, then weight after drying and calculate the yield (kg hm^-2^).

#### 2.4.2 Determination of contents of active constituents

##### 2.4.2.1 Determination of polysaccharide content

Content of *C. pilosula* polysaccharides was determined using the phenol-sulphuric acid method as described by Gao et al. (2019).

Serial standard solutions was prepared by adding 0, 0.2, 0.4, 0.6, 0.8, 1.0 mL of glucose standard solution (0.1 mg mL^-1^) into colorimetric tubes respectively, and topped up to 2 mL with distilled water. Then, 1 mL of 5% phenol solution was added, mixed well and 5 mL of concentrated sulphuric acid was added quickly. The mixture was blended well, heated in a boiling water bath for 15 minutes and cooled to room temperature (25°C). After calibrating using distilled water as the blank reagent, the absorbance of glucose solutions at 490 nm was recorded and the standard curve plotted.

To preparation of sample solutions, the *C. pilosula* powder (0.1 g) was accurately weighed and put into 100 mL erlenmeyer flask with stopper, mixed with 50 ml of 80% ethanol and heated in a water bath for 1 h. The extract was filtered while it was hot. The residue was rinced with hot 80% ethanol for 3 times and the eluents were pooled with the ethanol extract. The washed residue and filter paper were put into an erlenmeyer flask and extracted with 50 mL distilled water in a water bath at 90°C for 1 hour before the extract was filtered while it was hot. The residue and flask was washed with hot water and pooled. The ethanol and aqueous extracts were pooled and filled to 100 mL in a volumetric flask. Then, 1 mL *C. pilosula* extract was pipetted precisely into a colorimetric tube and followed the colorimetric method of standard solution and the content of polysaccharides was calculated according the standard curve.

##### 2.4.2.2 Determination of contents of lobetyolin, syringin and atractylenolide III

The contents of lobetyolin, syringin and atractylenolide III macrocephalae were determined by the method described by Xia et al. (2017).

(1) UPLC - MS/MS analysis.

The analysis was conducted on an ACQUITY UPLC BEH C18 column (2.1 mm × 50 mm, 1.7 μm; Waters Corporation, America, USA) at 30 °C at a flow rate of 0.4 mL min^-1^ and an injection volume of 2 μL. The mobile phase consisted of methanol (chromatographic pure) (A) and 0.1% ammonium formate aqueous solution (analytical pure ammonium formate, Watson’s distilled water) (B). Elution program was as follow: 0~0.5 min, 5% A; 0.5~5 min, 5~95% A; 5~7 min, 95% A; 7~7.1 min, 95~5% A; 7.1~9 min, 5% A.

(2) Preparation of standard solutions.

The standard substances of lobetyolin, syringin and atracylenolide III were accurately weighed and prepared as mixed control solution at concentrations of 10, 50, 100, 400, 600 and 1000 ng mL^-1^ with methanol (chromatographically pure).

(3) Preparation of sample solutions.

The *C. pilosula* powder (0.1 g) was accurately weighed into 100 mL erlenmeyer flask with stopper and mixed with 25 mL 75% methanol. The erlenmeyer flask was weighed, then extracted for 45 min at room temperature by ultrasonication. The flask was weighed again and make up the weight with 75% methanol. The sample was centrifuged (10000 r min^-1^) for 15 min, and the supernatant was collected and filtered with a nylon filter membrane (0.22 μm, 13 mm). The resulting supernatant was stored in a 1 mL sample bottle prior to UPLC-MS/MS analysis.

#### 2.4.3 Determination of photosynthetic characteristics

Photosynthetic characteristics were measured on August 19th 2022, September 5th 2022 and September 20th 2022. Five representative plants were randomly selected from each plot, and three fully expanded functional leaves with no damages in the upper and middle parts were selected from each plant for testing. Photosynthetic rate (Pn) of leaves was measured with a portable photosynthesizer (LI-6400XT, Licor Corporation, Lincoln, Nebraska, USA) on 9:00 - 11:00 a.m. SPAD value (chlorophyll) was measured in undamaged and intact functional leaves with a SPAD chlorophyll meter (SPAD-502).

#### 2.4.4 Determination of enzyme activities of leaves

Functional leaves of plants were collected and stored in liquid nitrogen for determination of metabolic enzyme activities. Nitrate reductase (NR), sucrose synthetase (SS) and sucrose phosphoric acid synthetase (SPS) activities were determined with kits purchased from Beijing Solarbio Science & Technology Co. Ltd (China). And phosphomannomutase (PMM) activity was determined with kits obtained from Shanghai Bohu Biotechnology Co. Ltd (China).

### 2.5 Data calculation

According to the yield and active constituent content, the active constituent yield was calculated as follows:

ACY=ACC × Y

where ACY (kg hm^-2^) is the active constituent yield, ACC (mg g^-1^) is the active constituent content, Y (kg hm^-2^) is the yield per unit area.

According to the active constituent yield and input costs, the output-input ratio was calculated as follows:

R=Y/(PC + FC)

where R (g $^-1^) is the output-input ratio, Y (g hm^-2^) is the active constituent yield, PC ($ hm^-2^) is the product annual cost, FC ($ hm^-2^) is the annual cost of spraying in the field.

### 2.6 Statistical analysis

Data presented in tables and figures are the mean of three replicates (n=3). Analysis of variance (ANOVA) and stepwise linear regression were performed with IBM SPSS Statistics 26.0 to compare the significance of differences between treatments at the 0.05 level. Origin 9.9 (Origin Lab, Corp., Northamp-ton, MA, USA) software was used to draw the figures.

## 3 Results

### 3.1 Effects of chlorocholine chloride and humic acid on root growth and yield of *C. pilosula*

**Figure 1:**
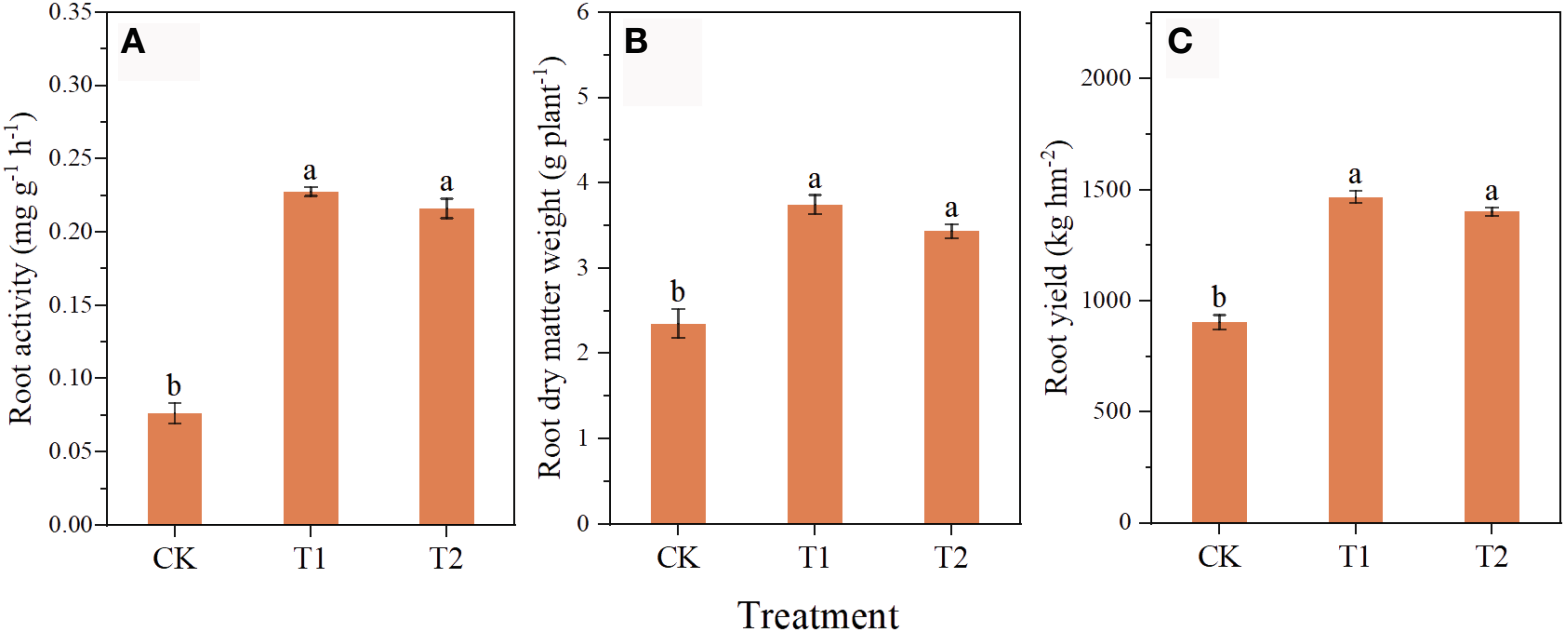
Effects of chlorocholine chloride and humic acid regulator on growth and yield of *Codonopsis pilosula*. Note: the root activity **(A)**; the root dry matter weight **(B)**; the yield **(C)**; All data represent mean ± standard error. Different lowercase letters indicate significant differences in each treatment *(P<0.05)*. Water treatment (CK), chlorocholine chloride treatment (T1), humic acid regulator treatment (T2).

The application of chlorocholine chloride and humic acid improved root growth of *C. pilosula* (**Figure 1**). Compared with CK, the root activity in T1 and T2 was significantly increased by 198.0 and 182.9%, the root dry matter weight by 51.4 and 46.1%, and the yield by 59.1 and 54.9%, respectively. There was no significant difference in root activity, root dry matter mass, and yields between T1 and T2.

### 3.2 Effects of chlorocholine chloride and humic acid on active constituents

**Figure 2:**
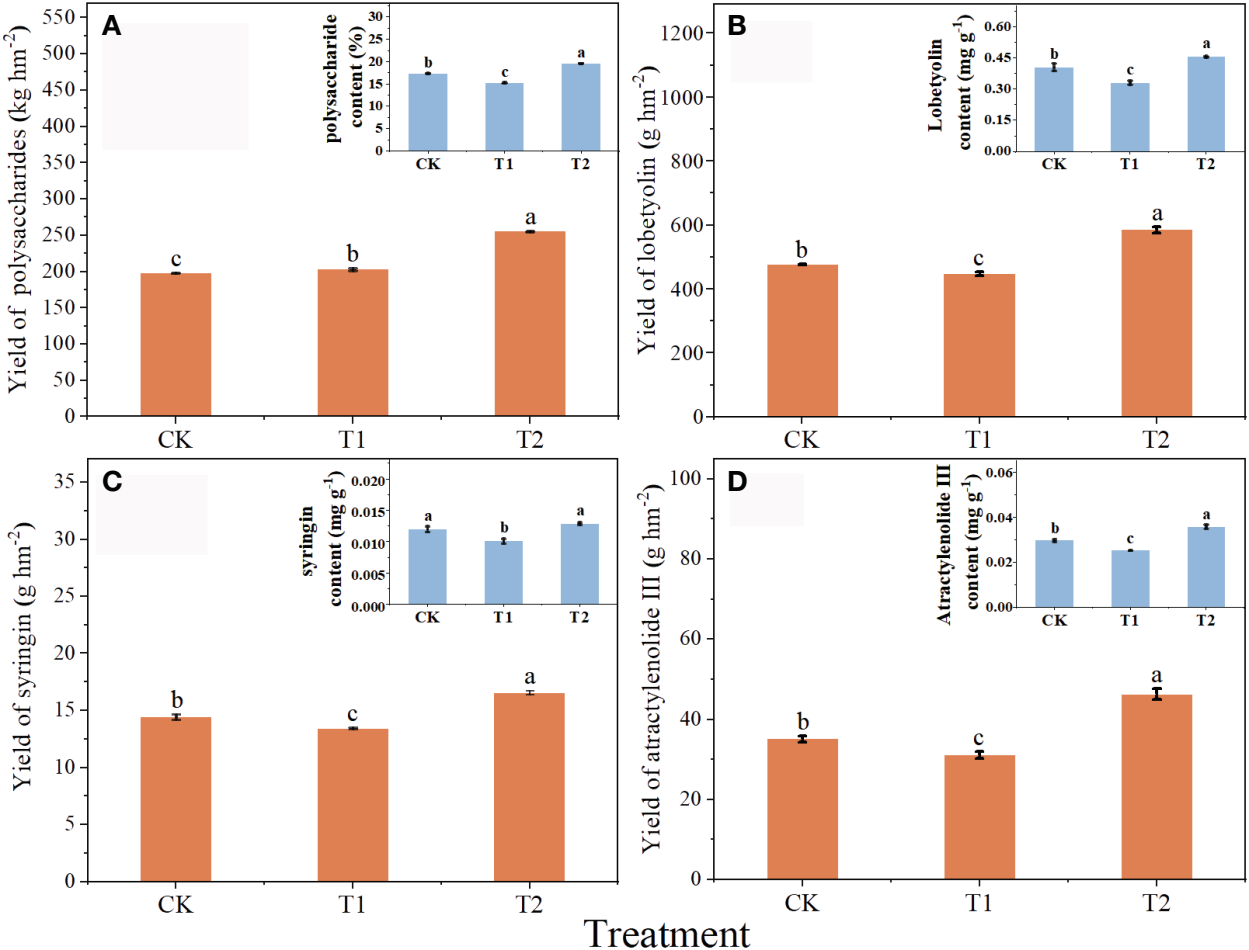
Effects of chlorocholine chloride and humic acid regulator on the quality of *Codonopsis pilosula*. **(A)** the yield of *Codonopsis pilosula* polysaccharides, **(B)** the yield of lobetyolin; **(C)** the yield of syringin; **(D)** the yield of atracylenolide III; and in the upper right corner is the corresponding active constituent content. All data represent mean ± standard error. Different lowercase letters indicated significant differences in the yield or content of active constituent for each treatment *(P<0.05*). Water treatment (CK), chlorocholine chloride treatment (T1), humic acid regulator treatment (T2).

Chlorocholine chloride was not conducive to the accumulation of active constituents in *C. pilosula*, while humic acid promoted the synthesis of active constituents (**Figure 2**). Compared with CK, the content of polysaccharides, lobetyolin, syringin and atractylenolide III in T1 was decreased by 13.7, 22.6, 19.1 and 17.8%. However, the content of *C. pilosula* polysaccharide, lobetyolin and atractylenolide III in T2 was increased by 12.7, 12.4 and 20.6%. The contents of active constituents showed significant difference between T1 and T2.

The yield of active constituents of *C. pilosula* was calculated to evaluate the effects of different treatments (**Figure 2**). Compared with CK, the yield of lobetyolin, syringin and atractylenolide III in T1 was significantly reduced by 6.3, 7.3 and 13.0%, respectively. However, the yields of *C. pilosula* polysaccharide, lobetyolin, syringin and atractylenolide III in T2 was significantly increased by 29.0, 22.8, 14.8 and 32.0%, respectively. The yield of active constituents in T2 were 25.8, 30.6, 23.2 and 49.2% higher than that in T1, respectively.

### 3.3 Effects of chlorocholine chloride and humic acid on photosynthetic characteristics and enzyme activities of leaves

Both chlorocholine chloride and humic acid increased the photosynthetic rate (Pn) and chlorophyll content (SPAD value) of *C. pilosula* (**Table 1**). The photosynthetic rate in T1 and T2 was significantly increased as compared with CK after each spraying application, while there was no significant difference between T1 and T2. After each spraying application, the SPAD value in T1 was significantly increased by 23.2, 18.4 and 15.5% respectively when compared with CK, while in T2 it was increased by 27.6, 18.2 and 8.0%, respectively.

**Table 1:** Effects of chlorocholine chloride and humic acid regulator on photosynthetic and metabolic enzyme activities of *Codonopsis pilosula*.

Period	Treatments	*Pn* (μmol m^-2^ s^-1^)	SPAD value	NR (μg g^-1^ h^-1^)	SS (mg g^-1^ h^-1^)	SPS (μg g^-1^ h^-1^)	PMM (μg g^-1^ h^-1^)
2022/8/19	CK	9.64 ± 0.26b	22.22 ± 0.26b	9.00 ± 0.82b	5.80 ± 0.17b	56.97 ± 3.87b	185.32 ± 5.79a
	T1	11.82 ± 0.73a	27.37 ± 043a	7.43 ± 0.75b	3.19 ± 0.46b	49.95 ± 2.39b	144.24 ± 3.47b
	T2	11.11 ± 0.26a	28.35 ± 0.80a	17.85 ± 1.07a	6.21 ± 0.25a	98.68 ± 3.03a	179.35 ± 2.03a
2022/9/5	CK	9.76 ± 0.38b	24.75 ± 0.32b	15.22 ± 0.30b	1.54 ± 0.35b	140.86 ± 6.35c	146.21 ± 2.36b
	T1	12.24 ± 0.59a	29.30 ± 0.36a	20.29 ± 2.69a	0.89 ± 0.14b	182.30 ± 2.55b	107.41 ± 2.49c
	T2	11.24 ± 0.51a	29.27 ± 0.44a	23.78 ± 0.74a	3.76 ± 0.06a	222.40 ± 6.64a	162.65 ± 1.91a
2022/9/20	CK	8.81 ± 0.09b	30.88 ± 0.65c	13.44 ± 0.50b	8.41 ± 0.39b	86.42 ± 0.46c	128.88 ± 0.69b
	T1	12.29 ± 0.64a	35.67 ± 0.47a	16.71 ± 0.44a	6.85 ± 0.35c	122.05 ± 1.33b	117.56 ± 1.22c
	T2	11.17 ± 0.45a	33.35 ± 0.64b	13.78 ± 0.38b	9.99 ± 0.28a	161.10 ± 1.78a	146.29 ± 1.59a

All data represent mean ± standard error. Different lowercase letters indicate significant differences between treatments over the same period (*P*<0.05). Pn (the photosynthetic rate); SPAD (chlorophyll content); NR (nitrate reductase); SS (sucrose synthetase); SPS (sucrose phosphoric acid synthetase); PMM (phosphomannomutase). Water treatment (CK), chlorocholine chloride treatment (T1), humic acid regulator treatment (T2).

After the first spraying application, the activities of SS and PMM in T1 were significantly reduced by 81.6 and 28.5% compared with CK, while the content of NR and SS in T2 were increased by 98.3 and 7.0%, respectively. After the second spraying application, the activities of NR and SPS in T1 were significantly increased by 33.3 and 29.4% compared with CK, the activities of SS and PMM were reduced by 73.1 and 24.4% compared with CK, while the activities of NR, SS, SPS and PMM in T2 were significantly increased by 54.6, 144.5, 57.9 and 11.2%, respectively. After the third spraying application, the activities of NR and SPS in T1 were increased and SS and PMM activities were reduced, while the activities of SS, SPS and PMM in T2 were significantly increased by 18.7, 86.4 and 13.5% compared with CK, respectively.

### 3.4 Economic benefits of spraying chlorocholine chloride and humic acid regulator

**Figure 3:**
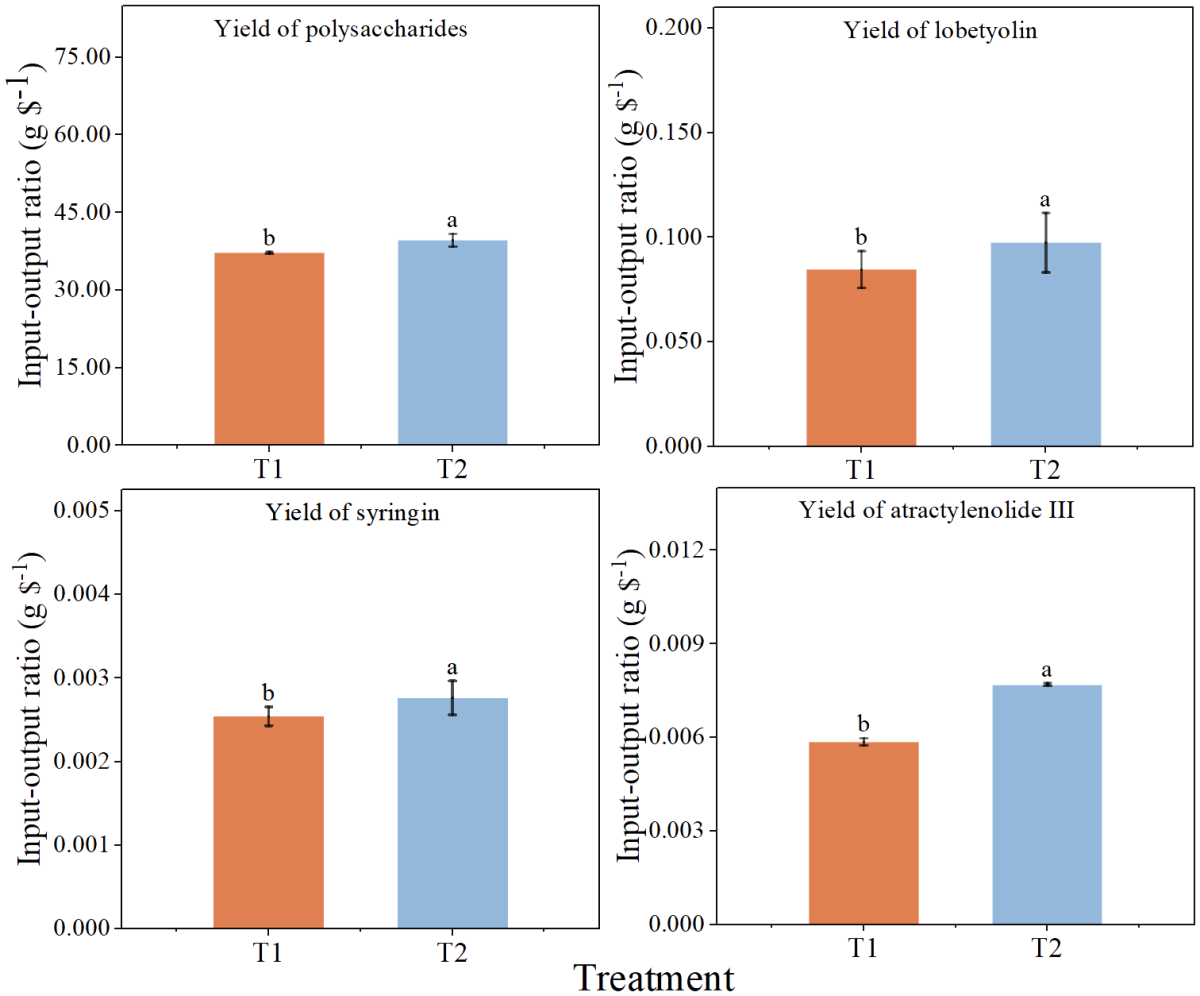
Economic benefit of spraying chlorocholine chloride and humic acid regulator. All data are mean ± standard error. Different lowercase letters indicated that the ratio of output to input of each treatment was significantly different (*P<0.05*). chlorocholine chloride treatment (T1), humic acid regulator treatment (T2).

Output-input ratio well reflects the economic benefits of each treatment. The total cost of chlorocholine chloride was 103.6 $ hm^-2^ and that of humic acid regulator was 117.6 $ hm^-2^ (**Table 2**). The output-input ratios based on the yield of the four active constituents in T2 were significantly higher than those in T1 by 6.5, 15.2, 8.7 and 31.2%, respectively (**Figure 3**), which indicate that economic benefits of humic acid regulator are higher than those of chlorocholine chloride.

**Table 2:** The cost of spraying chlorocholine chloride and humic acid regulator.

	Chlorocholine chloride	Humic acid regulator
The product annual cost ($ hm^-2^)	75.6	61.6
The annual cost of spraying in the field ($ hm^-2^)	28.0	56.0
Total cost ($ hm^-2^)	103.6	117.6

## 4 Discussion

### 4.1 Chlorocholine chloride and humic acid regulator increased the yield of *C. pilosula*

Due to the continuously growing market for traditional Chinese medicine, improving the yield and quality of cultivated *C. pilosula* is very important for meeting the rising market demand. This study found that foliar spraying of chlorocholine chloride and humic acid regulator on *C. pilosula* can promote root growth and development, and can both increase the yield significantly, which is similar to the finding in study of Bayat et al. (2021) and Jiang et al. (2023). Studies have found that photosynthesis is considered as the foundation of yield formation. The growth and development of plants, as well as their yield formation, are closely correlated with the accumulation and distribution of photosynthate (Sarker et al., 2020; Song et al., 2020). Study of Ren et al. (2011) and Fang et al. (2019) found that plant growth regulator can significantly increase the yield of plant by improve the SPAD and photosynthetic rate, which is consistent with our study. Ali et al. (2022) found that humic acid increased yield by regulating cell membrane permeability and then improving root absorption capacity. van Tol de Castro et al. (2021) found that humic acid can affect the synthesis of metabolic products in *Oryza sativa* by regulating photosynthesis, thereby stimulating the potential of plant growth. This study found that due to the different compositions of chlorocholine chloride and humic acid regulators, they may increase the yield of *C. pilosula* in different ways. Chlorocholine chloride may increase the yield by increasing photosynthetic rate and nitrate reductase activity (Huang et al., 2018; Afreen et al., 2023), while humic acid regulator may increase the yield by stimulating root activity, promoting nutrient uptake by root system and increasing activity of sucrose phosphate synthase (Haghighi et al., 2012; Ali et al., 2022).

**Figure 4:**
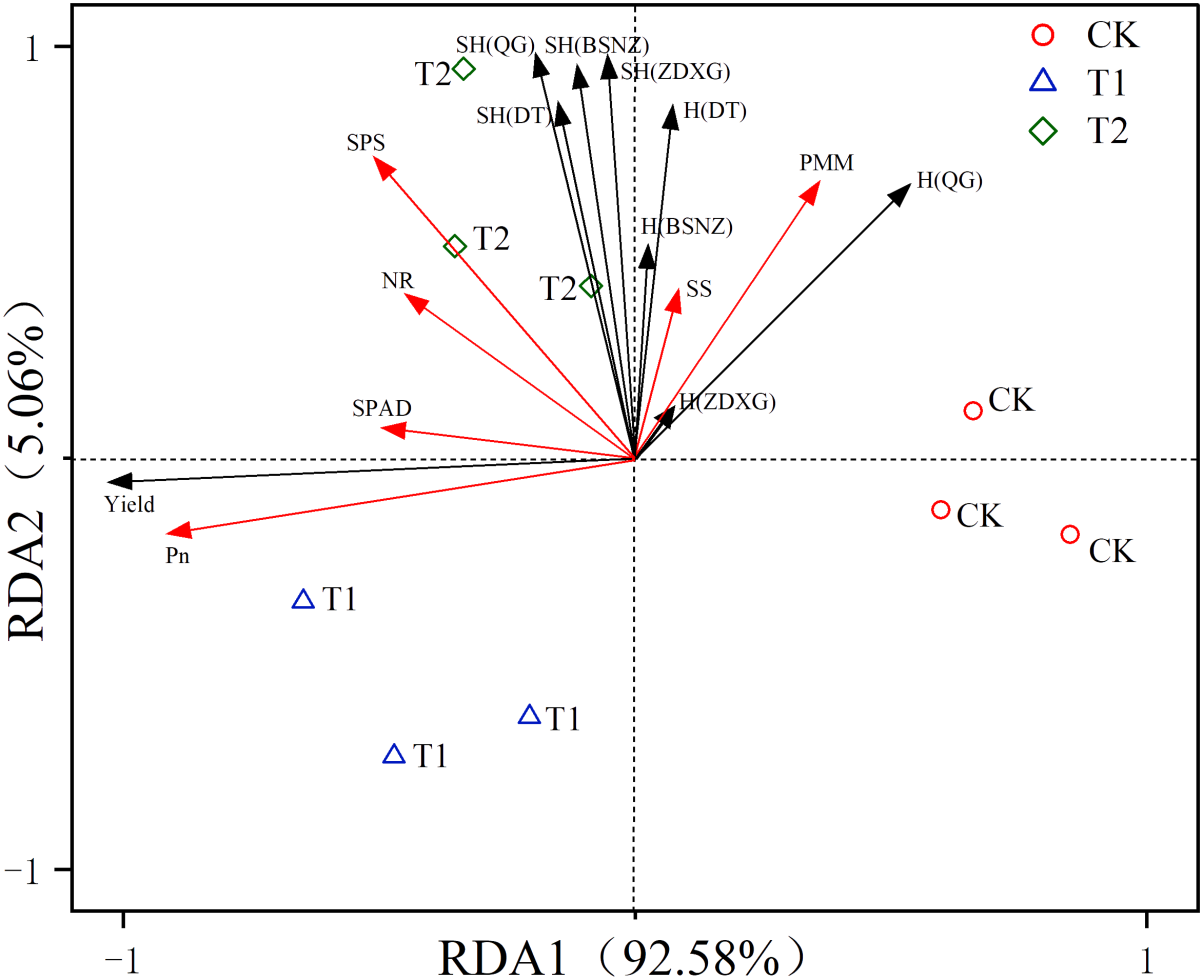
Redundant analysis of photosynthesis and metabolic enzyme activity versus yield and active constituent. Pn (the photosynthetic rate); SPAD (chlorophyll content); NR (nitrate reductase); SS (sucrose synthetase); SPS(sucrose phosphoric acid synthetase); PMM (phosphomannomutase); SH(DT) (the yield of *Codonopsis pilosula* polysaccharides); SH(QG) (the yield of lobetyolin); SH(ZDXG) (the yield of syingin); SH(BSNZ) (the yield of atracylenolide III); H(DT) (the *Codonopsis pilosula* polysaccharide content); H(QG) (the lobetyolin content);H(ZDXG) (the syringin content); H(BSNZ) (the atracylenolide III content).

Chlorophyll function is an intermediate carrier of photosynthesis and its content is a significant indicator of photosynthetic rate and leaf senescence (Biswal et al., 2012; Taiz et al., 2014). In this study, there were positive correlations between the SPAD value and Pn and the yield of *C. pilosula* (**Figure 4**). The significant difference in chlorophyll content was observed between chlorocholine chloride and humic acid regulator treatments in the later growing stage. However, there was no difference in the photosynthetic rate of the two treatments, because the photosynthetic rate might be no longer affected by the chlorophyll content once chlorophyll content reaches a certain threshold level (Li et al., 2024). At this time, there was no difference in the yield of the herb between the two treatments. Therefore, photosynthetic rate is the key factor affecting yield, which is consistent with the results of linear regression analysis in this study (**Table 3**).

**Table 3:** Stepwise linear regression photosynthesis and metabolic enzyme activity on *Codonopsis pilosula* yield and quality.

Dependent variable	Regression equation	R^2^	*P*
Yield	y=-996.65 + 197.48x_1 + _0.94x_2_	0.975	0.009
*Codonopsis pilosula* polysaccharide content	y=11.55 + 0.04x_3_	0.448	0.049
Lobetyolin content	y=0.96-0.05x_1_	0.469	0.042
Atracylenolide III content	y=0.01 + 0.05e^-4^x_2 + _0.09e^-4^x_3_	0.805	0.030
Yield of *Codonopsis pilosula* polysaccharide	y=194.53 + 0.18x_2_	0.496	0.034
Yield of lobetyolin	y=239.06+0.65x_2 + _1.31x_3_	0.867	0.011
Yield of syringin	y=9.02 + 0.01x_2 + _0.03x_3_	0.827	0.018
Yield of atracylenolide III	y=10.26 + 0.07x_2 + _0.13x_3_	0.814	0.026

x_1_ is the photosynthetic rate; x_2_ is sucrose phosphoric acid synthetase activity; x_3_ is phosphomannomutase activity.

### 4.2 Chlorocholine chloride reduced the yield of active constituents but humic acid regulator acted contrary

Active constituents are secondary metabolites produced during the growth and development of medicinal plants, which are also the key substances to exert medicinal effectiveness (Li et al., 2020). At present, the root yield and the contents of active constituents are the criteria for identifying the quality of medicinal plants in market, this results in a narrow and lopsided judgement. Therefore, the concept of active constituents yield was introduced here to comprehensively evaluate the quality of medicinal plants by linking the yield to the active constituent content. In this study, chlorocholine chloride led to a significant decrease in the yields of various active constituents in *C. pilosula* compared with CK, while humic acid regulator increased them. This is similar to the finding of Shah et al. (2018) and Afreen et al. (2023), it was because that chlorocholine chloride and humic acid regulator can affect active constituent content by regulating the related enzymes and expression of enzyme genes involved in the synthesis of active constituents.

Carbon metabolism is a fundamental metabolic process affecting plant growth and development, therefore metabolism enzymes are closely related to metabolites of plants (Banzouzi Bouzika et al., 2022). In this study, metabolism enzymes like sucrose synthetase, sucrose phosphoric acid synthetase and phosphomannomutase showed significant and positive correlations with the yields of four active constituents in *C. pilosula* (**Figure 4**), among which sucrose phosphoric acid synthetase and phosphomannomutase were the main factors affecting synthesis of active constituents (**Table 3**). It may because sucrose synthetase and sucrose phosphoric acid synthetase were two key enzymes in carbon metabolism (Banzouzi Bouzika et al., 2022). When their enzyme activity was high, they accelerate the synthesis and decomposition of photosynthetic products, providing more carbon frameworks for the synthesis of organic compounds (Galtier et al., 1995; Liu et al., 2024). It has indicating that carbon metabolism affected the synthesis and accumulation of these active constituents.

Studies have shown that sucrose synthetase facilitates the reversible decomposition of sucrose into glucose and fructose (Li et al., 2021). Phosphomannomutase enzyme is the key enzyme for GDP-mannose synthesis. GDP-mannose plays a role in synthesizing the sugar chain which is the starting material for the synthesis of carbon-based active constituents (Cao et al., 2020). Our results showed that both chlorocholine chloride and humic acid regulator could enhance the activity of sucrose phosphoric acid synthetase and subsequently provide more precursors for the synthesis of active constituents, but the effects of chlorocholine chloride and humic acid regulator on the activities of sucrose synthetase and phosphomannomutase were different. Chlorocholine chloride caused reduced activities of sucrose synthetase and phosphomannomutase, whereas humic acid regulator enhanced those. Thus, the differences in the yield of active constituents between chlorocholine chloride and humic acid regulator could be attributed to their different effects on enzymes such as sucrose synthetase and phosphomannomutase.

### 4.3 Humic acid regulator had better economic benefits than chlorocholine chloride in the cultivation of *C. pilosula*

The pharmacological effect of medicinal plants in treating human illnesses relies on their active constituents (Li et al., 2020). Previous study had evaluated the quality of medicinal plants by their active constituent content (Wang et al., 2019; Shen et al., 2021), and evaluated the economic benefits according to the yield of raw heral material available in the market. Active constituent yield proposed in this study may be a more scientific and reasonable indicator to combine agricultural production with practical medical applications. Chlorocholine chloride is a common growth regulator, its price is lower than that of humic acid regulator due to its industrialized production (Rademacher, 2015). Therefore, the economic benefits of using chlorocholine chloride and humic acid regulator by foliar spraying were comprehensively evaluated to determine the possibility of substituting chlorocholine chloride with humic acid regulator from an economic perspective. In this work, output-input ratios based on the yield of four active constituents in two treatments were calculated, the results indicate that output-input ratios of humic acid application were higher than those of chlorocholine chloride. The greater improving effect of humic acid regulator on active constituent yield compensated for the increased input costs, this resulted in that humic acid regulator demonstrated better application benefits than chlorocholine chloride. Furthermore, with the industrialization and commercialization of humic acid regulator, its price is expected to drop and bring greater economic benefits. Furthermore, previous study has indicated that chlorocholine chloride has slight toxic properties and a prolonged degradation period (Wang and Hao, 2023), which may result in residues in medicinal plants and soil and affect the environment and plants adversely (Zhao et al., 2018; Sutcharitchan et al., 2020). By contrast, humic acid regulator was made from raw materials that are widely available in nature (Shah et al., 2018), making it an environmentally friendly and non-polluting product. Therefore, replacing chlorocholine chloride with humic acid regulator is a feasible measure in the cultivation of *C. pilosula*.

## 5 Conclusion

Both chlorocholine chloride and humic acid regulator increased the root yield of *C. pilosula*. But chlorocholine chloride was adverse to the accumulation of active constituents, while humic acid regulator promoted the yield of active constituents achieving a win-win goal. In addition, humic acid regulator increased yield by increasing the chlorophyll content and photosynthesis of *C. pilosula*, and promote the accumulation of active constituents by increasing the activity of sucrose phosphoric acid synthetase and phosphomannomutase enzymes. The application of humic acid regulator had higher economic benefits than chlorocholine chloride. In summary, humic acid regulator can be applied as a substitute for chlorocholine chloride in the cultivation of *C. pilosula* by maintaining root yield, increasing active constituents yields and improving economic benefits.

## Data availability statement

The raw data supporting the conclusions of this article will be made available by the authors, without undue reservation.

## Author contributions

GH: Data curation, Formal analysis, Writing – original draft. HM: Data curation, Formal analysis, Writing – original draft, Investigation. YC: Investigation, Visualization, Writing – original draft. KW: Funding acquisition, Software, Writing – review & editing. QZ: Funding acquisition, Conceptualization, Methodology, Writing – review & editing. ZY: Conceptualization, Funding acquisition, Methodology, Writing – review & editing.
